# Case Report: Revision surgery for a missed posterior dislocation of the humeral head in a shoulder fracture-dislocation and literature review

**DOI:** 10.3389/fsurg.2026.1812315

**Published:** 2026-04-30

**Authors:** Xin Hu, Gang Zheng, Wei Zhao, Hao Dong, Jinhao Li, Zhe Hu, Haoming Lu, Dewei Shen, Nan Wang

**Affiliations:** 1 Department of Spine Surgery, Central Hospital Affiliated to Shenyang Medical College, Shenyang, Liaoning, China; 2 Department of Foot and Ankle Surgery, Central Hospital Affiliated to Shenyang Medical College, Shenyang, Liaoning, China

**Keywords:** case report, posterior shoulder dislocation, proximal humerus fracture, reverse Hill–Sachs lesion, revision surgery

## Abstract

**Background:**

Posterior shoulder dislocation is uncommon and therefore prone to being missed. When accompanied by a proximal humeral fracture, complex fracture lines may obscure the radiographic signs of dislocation. Inadequate standardization of the imaging workup may consequently result in inappropriate management and treatment failure.

**Case presentation:**

We report a young patient in whom posterior shoulder dislocation was missed at the initial assessment because axillary and scapular Y-view radiographs were not obtained and the available imaging was insufficiently interpreted. The dislocation persisted after the index operation. Revision surgery was performed on postoperative day 9 and resulted in a favorable 5-year outcome, with no radiographic evidence of humeral head avascular necrosis or post-traumatic osteoarthritis and near-complete recovery of shoulder function.

**Conclusion:**

Occult posterior shoulder dislocation should be strongly suspected in patients with complex proximal humeral fractures after high-energy trauma. Obtaining axillary or scapular Y-view radiographs, together with a systematic CT-based assessment, is essential. Even after early treatment failure, targeted revision surgery combined with structured rehabilitation may still achieve a satisfactory long-term outcome in young patients.

## Introduction

Posterior shoulder dislocation (PSD) is a relatively rare injury, accounting for 1.1%–4.7% of all shoulder dislocations (1–3). Because the clinical presentation is nonspecific and routine anteroposterior (AP) radiographs may show only subtle findings, the missed diagnosis rate at initial presentation can be as high as 60% (3, 4). Although seizures and electrical injury are traditionally associated causes (1, 2), PSD is increasingly encountered in the setting of high-energy trauma (5).

When PSD is combined with a proximal humerus fracture (PHF), diagnosis becomes more difficult (6, 7). Complex fracture lines often obscure radiographic signs of dislocation, resulting in misdiagnosis as isolated fractures and subsequent inappropriate management (4, 6). In addition, after dislocation, the humeral head is impacted against the posterior glenoid rim, and an anteromedial compression defect of the humeral head—termed a reverse Hill–Sachs lesion (RHSL)—is common and may substantially increase joint instability (2, 8).

To date, evidence-based diagnostic and treatment guidelines for this triad of injuries are lacking. Management is guided by the size of the RHSL and the duration of dislocation; surgical options include closed reduction, open reduction and internal fixation (ORIF), defect reconstruction with bone grafting, modified McLaughlin procedures, and shoulder arthroplasty (2, 9–11). Missed diagnosis or inappropriate management may result in persistent dislocation, osteonecrosis, and severe, permanent functional impairment (1, 5, 12). Here, we report a young patient whose postoperative computed tomography (CT) demonstrated a persistent posterior fracture-dislocation; timely revision surgery resulted in a satisfactory long-term outcome, and this report highlights practical points to help reduce missed posterior shoulder fracture-dislocations.

## Case presentation

### Patient information

A 36-year-old man presented to the emergency department with severe right shoulder pain and limited range of motion after a bicycle fall. He denied any history of seizures, electrical injury, alcohol misuse, or osteoporosis. He had no known chronic medical conditions and no prior surgery on the right shoulder. He reported no regular medication use and no known drug allergies. His family history was negative for epilepsy, recurrent shoulder instability, or metabolic bone disease. He was employed and independent in activities of daily living, and he denied tobacco or illicit drug use.

### Initial clinical findings

Physical examination revealed swelling and marked tenderness of the right shoulder. The affected limb was maintained in adduction and internal rotation.

### Imaging findings

**Figure 1:**
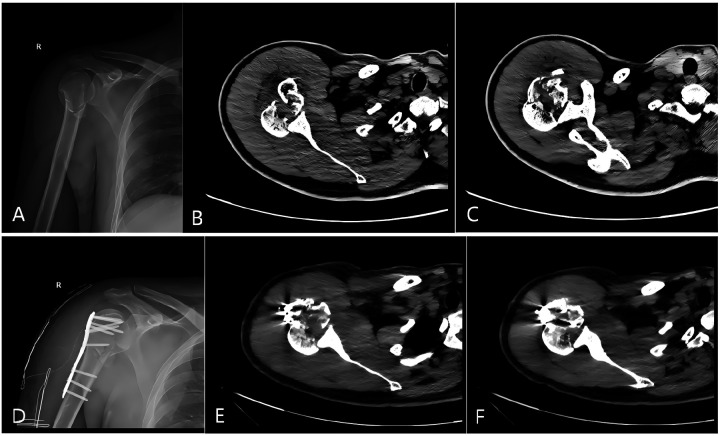
**(A)** Anteroposterior radiograph at the initial presentation shows a proximal humeral fracture with the “lightbulb sign”. **(B,C)** CT scans obtained at the initial presentation demonstrate a four-part proximal humeral fracture with loss of humeral head–glenoid congruency, a locked posterior dislocation of the humeral head, and a RHSL. **(D)** Anteroposterior radiograph after the initial surgery still shows the “lightbulb sign”. **(E,F)** Postoperative CT scans show poor humeral head–glenoid alignment, and the locked posterior dislocation was not corrected.

Although initial anteroposterior (AP) radiographs and CT were obtained (Figures 1A–C), the initial assessment focused mainly on the AP radiographs, and the axial CT images were not systematically reviewed to assess glenohumeral congruency. Standard trauma-series views (axillary or scapular Y) were not obtained, limiting direct assessment of the glenohumeral relationship. Because the posterior dislocation was not recognized preoperatively, the index procedure was planned mainly for fracture fixation. The first operation was performed through a deltoid-splitting approach, which provided limited visualization in this complex fracture-dislocation and did not allow the posteriorly locked humeral head to be adequately assessed intraoperatively. Consequently, concentric reduction of the glenohumeral joint was not achieved during the index operation.

Postoperatively, the patient reported shoulder pain and restricted passive range of motion, particularly external rotation. Follow-up radiographs and CT demonstrated persistent posterior dislocation of the humeral head relative to the glenoid (Figures 1D–F), indicating that the initial procedure did not address the underlying glenohumeral dislocation (Table 1).

**Table 1:** Timeline of the clinical course.

Time point	Setting	Key findings	Imaging	Intervention	Outcome/notes
Day 0 (injury)	Emergency department	Bicycle fall; severe right shoulder pain; limited ROM; arm held in adduction and internal rotation; full examination not tolerated	AP radiographs and CT obtained	—	Posterior shoulder dislocation was not recognized initially; no axillary or scapular Y view was obtained
Day 0–1 (initial management)	Operating room	Managed as a proximal humerus fracture	No intraoperative axillary fluoroscopy	Primary surgery: ORIF of proximal humerus via deltoid-splitting approach (as reported)	Postoperative pain and restricted passive ROM, particularly external rotation
Early postoperative period	Ward	Persistent pain and limited motion	Postoperative radiographs and CT	—	Imaging demonstrated persistent posterior glenohumeral dislocation
Postoperative day 9 (revision)	Operating room	Persistent locked posterior fracture-dislocation confirmed intraoperatively; RHSL present	Intraoperative fluoroscopy; postoperative 3D CT	Revision via a deltopectoral approach: removal of initial hardware; open reduction of the posterior dislocation; elevation and support of the humeral head defect with allograft; screw fixation of the defect; temporary K-wire stabilization; PHILOS plate fixation; capsular repair; and rotator cuff sutures tied through the plate	Post-revision CT confirmed anatomic fracture reduction and restoration of concentric glenohumeral alignment
0–3 weeks post-revision	Rehabilitation	Immobilization phase	—	External-rotation brace at ∼30° abduction and ∼30° external rotation; active hand/wrist exercises; elbow ROM exercises	Protection of the repair and fixation
3–6 weeks post-revision	Rehabilitation	Early mobilization phase	—	Sling; initiation of passive shoulder ROM with gradual progression	ROM gradually improved
≥6 weeks post-revision	Rehabilitation	Strengthening phase	—	Discontinue sling; progressive strengthening and loading	Functional recovery progressed
1 year post-revision	Follow-up visit	No pain; ROM: FF 170°, Abd 170°, ER 40°, IR 60°; ASES score 81	Radiographs showed union with no AVN	Routine implant removal	Continued recovery
5 years post-revision	Final follow-up	No pain; ROM: FF 180°, Abd 180°, ER 45°, IR 70°; ASES score 90	Imaging showed no AVN and no post-traumatic arthritis	—	Satisfactory long-term outcome

FF, forward flexion; Abd, abduction; ER, external rotation; IR, internal rotation; AVN, avascular necrosis; ORIF, open reduction and internal fixation; RHSL, reverse Hill–Sachs lesion.

### Revision surgery (treatment intervention)

Nine days after the initial procedure, revision surgery was performed because of the imaging findings and joint instability. The revision goals were to achieve concentric glenohumeral reduction, reconstruct the reverse Hill–Sachs defect, and obtain stable fixation of the fracture fragments to allow functional rehabilitation. Because the index operation had been performed through a deltoid-splitting approach and the posterior dislocation had been missed, revision was planned through a deltopectoral approach, which provides clearer exposure for complex proximal humeral fractures associated with dislocation. Under general anesthesia, the patient was placed in the beach-chair position. With the shoulder internally rotated, the previously implanted plate and screws were exposed and removed intact.

**Figure 2:**
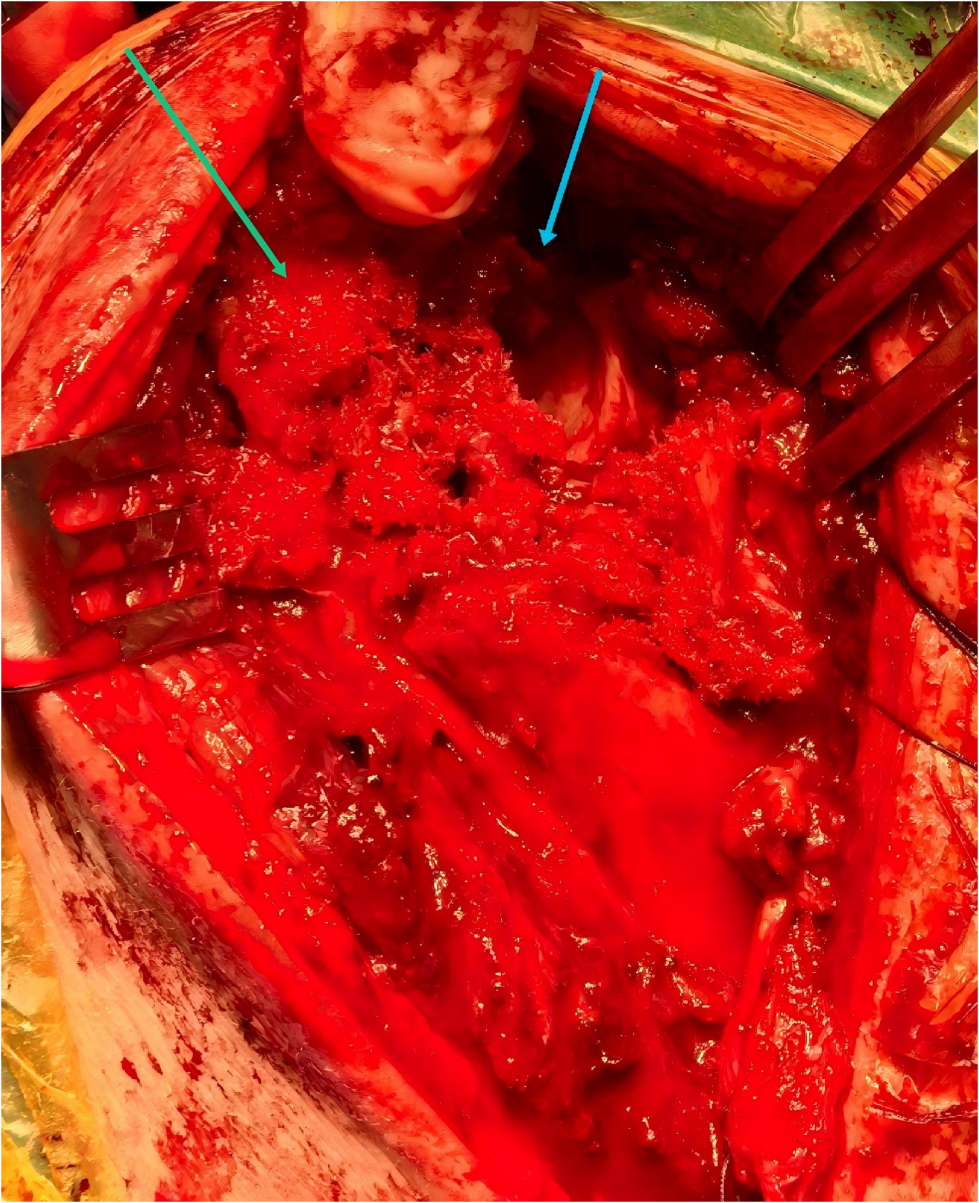
Intraoperative findings during revision surgery show glenohumeral malalignment, with the humeral head locked posteriorly behind the glenoid. The blue arrow indicates the glenoid, and the green arrow indicates the greater tuberosity of the humeral head.

Because this was a four-part fracture involving the lesser tuberosity, the fracture line of the lesser tuberosity was identified with care taken to protect the subscapularis, and the lesser tuberosity was mobilized along the fracture line together with the subscapularis tendon. No additional lesser tuberosity osteotomy was performed. The glenohumeral joint was then exposed by retracting the lesser tuberosity-subscapularis unit medially and the proximal humerus laterally, revealing incongruent alignment, with the humeral head fragment locked posterior to the glenoid (Figure 2).

A Kirschner wire was inserted into the humeral head through the fracture gap. A periosteal elevator was then introduced into the glenohumeral joint to lever the humeral head anteriorly and reduce it from its locked position posterior to the glenoid. Under direct visualization, the humeral head articular surface was anatomically reduced. Allograft bone was used to buttress the collapsed area, followed by screw fixation.

**Figure 3:**
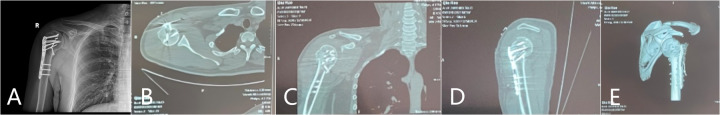
**(A)** Anteroposterior radiograph after revision surgery shows resolution of the “lightbulb sign,” with good humeral head–glenoid congruency, restoration of the neck–shaft angle, and satisfactory greater tuberosity height. **(B–E)** CT scans demonstrate anatomic fracture reduction, restoration of glenohumeral alignment, and screw buttressing of the RHSL.

A Proximal Humerus Internal Locking System (PHILOS) plate was then positioned and secured. Because PHILOS screws could not effectively control the superior aspect of the greater tuberosity, the posterior aspect of the greater tuberosity, or the lesser tuberosity, supplementary suture fixation was used in a parachute configuration. The subscapularis was sutured anteriorly at the tendon-bone junction, the supraspinatus superiorly, and the infraspinatus and teres minor posteriorly; the joint capsule was additionally sutured from anterior to posterior. The suture ends were then passed through the small peripheral holes of the plate and tied securely, creating a force distribution resembling the cords of a parachute. After reconstruction, the lesser tuberosity was reduced and fixed *in situ*. Medial displacement was not performed because it would have compromised fracture-surface matching, adversely affected bony healing, and increased subscapularis tension, thereby increasing the risk of fixation failure. Fluoroscopy confirmed fracture reduction, appropriate plate height, and restoration of glenohumeral congruency. Post-revision CT in the axial, coronal, and sagittal planes showed anatomical fracture reduction, restoration of the concentric glenohumeral relationship, and screw support of the RHSL (Figures 3A–E).

### Postoperative rehabilitation and follow-up

After revision surgery, the shoulder was immobilized for 3 weeks in an external brace at 30° of abduction and 30° of external rotation. During the first 3 weeks, the patient performed active finger and wrist exercises and routine elbow flexion–extension. At 3 weeks postoperatively, the brace was replaced with a sling, and passive range-of-motion (ROM) exercises were initiated. Active shoulder exercises were also initiated and progressed gradually as tolerated. At 6 weeks postoperatively, the sling was discontinued and gradual strengthening with progressive loading was initiated.

**Figure 4:**
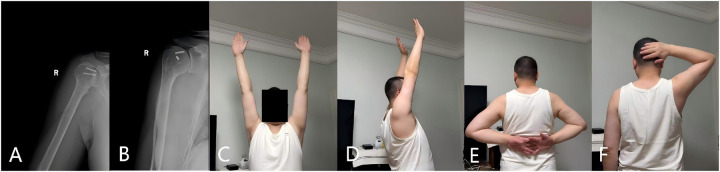
**(A,B)** Radiographs obtained 1 year after revision surgery show complete fracture union. The plate and several screws were removed; two screws were retained because they were located within the joint capsule. No radiographic signs of humeral head osteonecrosis are present. **(C–F)** At 1 year postoperatively, shoulder function had recovered well.

At 1 year postoperatively, the patient reported no pain and had good active shoulder ROM: forward flexion of 170°, abduction of 170°, external rotation of 40°, and internal rotation of 60°. The American Shoulder and Elbow Surgeons (ASES) score was 81 (Figures 4A–F), and planned hardware removal was performed.

**Figure 5:**
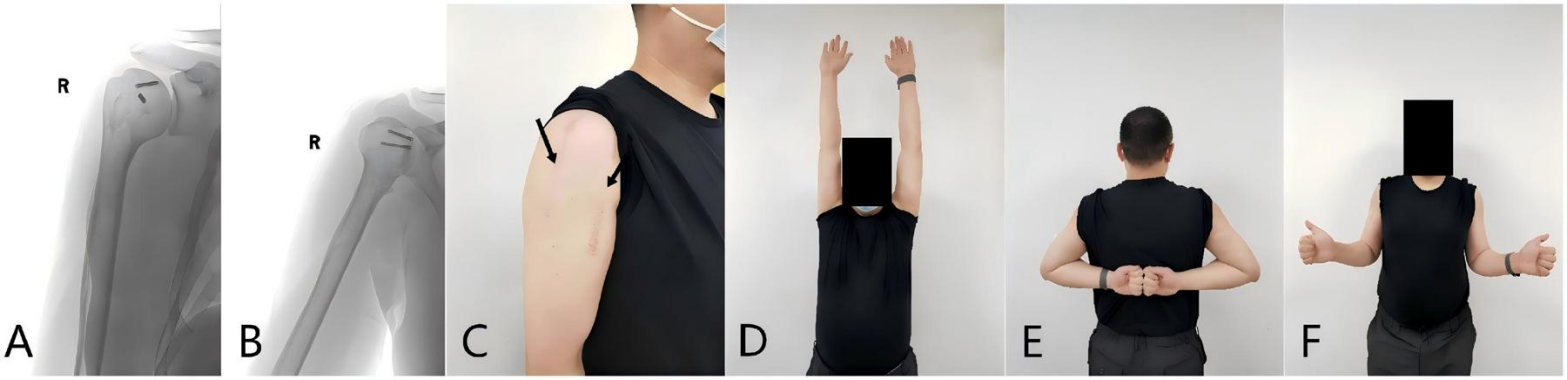
**(A,B)** Radiographs obtained 5 years after revision surgery show no radiographic signs of humeral head osteonecrosis. **(C)** The two black arrows indicate the incisions from the two procedures: the deltoid-splitting approach and the deltopectoral approach. **(D–F)** At 5 years after revision surgery, shoulder function had recovered to an excellent level.

At the 5-year follow-up, the patient reported no pain. Active shoulder ROM was excellent, with forward flexion of 180°, abduction of 180°, external rotation of 45°, and internal rotation of 70°. The ASES score was 90. Follow-up imaging showed no radiographic evidence of humeral head avascular necrosis (AVN) or post-traumatic arthritis (Figures 5A–F).

## Discussion

### Diagnostic pitfalls and imaging lessons

PSD is often described as a “diagnostic trap” in orthopaedic trauma emergencies (1) because its presentation is nonspecific and key findings on routine anteroposterior (AP) radiographs can be subtle, resulting in initial missed diagnosis rates of up to 60% (3, 4). In this case, the patient developed severe right shoulder pain and limited range of motion after high-energy trauma. On examination, the right arm was held in adduction and internal rotation. However, because of severe fracture-related pain and joint instability, the patient was unable to cooperate with the examination. Passive external rotation—one of the more characteristic findings—could not be assessed reliably during the initial emergency evaluation; thus, the diagnosis relied on comprehensive imaging. Accordingly, amid severe pain and complex fracture lines, clinicians may focus on the PHF itself, and the concomitant shoulder dislocation can be overlooked. Previous studies have also highlighted the phenomenon of “attention captured by the fracture while overlooking the dislocation” (4, 6).

A lack of standardized imaging assessment contributed to the missed diagnosis. With the widespread use of 3D CT reconstructions, many clinicians focus primarily on the reconstructed views, and axial images may be overlooked. Axial CT is more sensitive for posterior dislocation of the humeral head, whereas 3D reconstruction may be less revealing, particularly when 360° reconstructions are not provided by some institutions. In addition, inadequate standardized training among the initial clinicians led to failure to obtain axillary view or scapular Y view radiographs according to trauma protocols. This was a proximate technical contributor to the missed diagnosis in this case.

In addition, axial CT and 3D reconstructions served two purposes in this case. On the one hand, in suspected PSD or complex fracture-dislocation, CT helps delineate fracture morphology, bone defects, and joint alignment to guide treatment strategy (13). On the other hand, in this case, 3D CT reconstructions were used after revision surgery to confirm anatomic fracture reduction and restoration of a concentric glenohumeral relationship. They also demonstrated screw support of the RHSL.

Finally, from a practical clinical perspective, PSD combined with PHF is often described as a “diagnostic trap” not only because it is rare, but also because it tends to arise when imaging is incomplete, clinical suspicion for PSD is low, and attention is disproportionately directed to complex fracture lines. In this case, the initial emergency assessment relied on AP-only radiography, without axillary view or scapular Y view radiographs and without systematic review of the axial CT images; consequently, a locked posterior dislocation persisted after the initial surgery. Revision surgery was undertaken only after early postoperative imaging demonstrated poor glenohumeral alignment. These observations suggest that, when evaluating complex PHF, clinicians should explicitly aim to exclude occult PSD and include axillary view or scapular Y view radiographs, along with axial CT and CT-based 3D reconstructions, in the initial workup.

### Injury mechanism and clinical significance of RHSL

RHSL is an anteromedial compression defect of the humeral head formed by impaction against the posterior glenoid rim in the setting of PSD, essentially reflecting a specific contact pattern between the humeral head and the glenoid rim during posterior dislocation. Accordingly, it is more common in posterior fracture-dislocations and represents an important structural hallmark of this injury (2, 8). If RHSL is not recognized and incorporated into the treatment plan, residual structural instability may persist (2, 8).

The significance of RHSL lies not only in explaining the injury mechanism but also in its close association with treatment selection. The literature suggests that defect size is closely linked to the treatment approach, and joint-preserving repair often needs to be individualized based on defect severity and bone quality (2, 8, 14). Previous reports have described anatomical reconstruction using autograft or allograft cancellous bone, with good-to-excellent functional scores and a reduced risk of recurrent dislocation (14). Autograft reconstruction of RHSL combined with capsular repair has also been reported to yield satisfactory function at long-term follow-up (8). In addition, for locked posterior dislocation with defects of approximately 30%–50%, fresh-frozen osteochondral allograft reconstruction has been reported to yield good mid-term outcomes (15). Collectively, these studies converge on a common rationale: once the RHSL exceeds a certain threshold, reduction or fracture fixation alone may be insufficient to restore stable glenohumeral congruence. Concomitant management of the bony defect, together with reinforcement of stabilizing structures, may be required to reduce the risk of persistent posterior dislocation or redislocation (2, 8, 14, 15).

In this case, revision surgery aimed to restore glenohumeral congruence and address the defect. This approach is consistent with strategies described in the literature that prioritize joint preservation through defect reconstruction and repair of stabilizing structures (8, 14). In young, high-demand patients, it may help preserve long-term function while avoiding arthroplasty. In the present case, persistent posterior dislocation after the initial treatment was corrected with timely revision surgery. Structural bone support and fixation were applied at the RHSL impaction site as part of defect reconstruction. Stabilizing structures, including the capsule and rotator cuff, were also repaired. Ultimately, long-term stability and good function were achieved.

### Treatment strategy: ORIF and revision within a humeral head–preserving approach

In managing shoulder dislocation with concomitant PHF and RHSL, treatment typically has three goals: reduction of the dislocation, stabilization of the fracture, and management of the articular surface defect. Selection of the surgical approach requires balancing injury acuity (timing), defect size, fracture pattern, and the patient's functional demands.

In young patients with high functional demands, prior studies suggest that humeral head preservation, together with restoration of anatomic alignment and joint stability, should be a key goal (2, 6, 14). Robinson et al. proposed a strategy for complex posterior fracture-dislocations that includes open reduction, bone grafting of humeral head defects when indicated, and fracture fixation. They reported acceptable functional outcomes but emphasized vigilance for complications such as AVN (6). Similarly, case series using locked-plate ORIF for locked posterior fracture-dislocations reported good function, although AVN may still introduce uncertainty into outcomes (5). Accordingly, early and accurate diagnosis and timely, targeted surgical management (including defect reconstruction and repair of stabilizing soft tissues) are important for improving prognosis (1, 5, 6).

The modified McLaughlin procedure and its variants are frequently employed to manage chronic or missed locked posterior dislocations associated with RHSL. Published reports include case reports in which these procedures were used as salvage options (16), as well as case series reporting high UCLA scores and low recurrence when they were combined with bone grafting (9). Another report described autograft reconstruction of the humeral head defect after open reduction. At 5-year follow-up, the patient achieved a perfect Constant score, supporting the feasibility of a joint-preserving strategy with defect reconstruction (8). As defect severity increases, published reports suggest that treatment should be tailored to the extent of humeral head articular surface involvement. When the defect involves >50% of the articular surface, osteochondral transplantation or shoulder arthroplasty has been recommended, with the choice between hemiarthroplasty and total shoulder arthroplasty individualized by age and activity level (3). Therefore, joint preservation and arthroplasty should not be viewed as opposing concepts, but rather as distinct stages within a treatment algorithm determined by defect size and reconstructability.

These treatment pathways align closely with the clinical course and outcome in the present case. Because the initial emergency imaging assessment was incomplete, PSD with PHF was misinterpreted as an isolated fracture. In addition, the index procedure was performed through a deltoid-splitting approach, and because the posterior dislocation was missed, the surgical focus was directed solely toward fracture fixation. Persistent posterior dislocation and intra-articular malalignment after the initial ORIF suggested that the dislocation had not been corrected. Based on early postoperative imaging evidence of failed reduction, revision surgery was performed on postoperative day 9. Intraoperatively, PSD with a comminuted fracture and RHSL was confirmed; the defect involved approximately 25% of the articular surface. The revision focused on restoring glenohumeral alignment via open reduction. The depressed fragment was elevated and secured with screws, and allograft bone was used to buttress the collapsed area. A PHILOS locking plate was used for fracture fixation, and stability was reinforced with capsular repair and rotator cuff fixation using a parachute technique. Postoperative CT in the axial, coronal, and sagittal planes confirmed anatomic fracture reduction, restoration of a concentric glenohumeral relationship, and adequate screw support for the defect.

At follow-up, the patient achieved solid union and functional recovery after structured rehabilitation, with no pain through 5 years and no imaging evidence of AVN or post-traumatic arthritis. Given the reported risk of AVN after ORIF (5, 6), this case suggests that a targeted revision ORIF—aimed at restoring glenohumeral congruency, reconstructing the humeral head defect, and reinforcing stabilizing structures—may still achieve satisfactory long-term outcomes in a young patient, even after a missed diagnosis necessitates revision. These observations may help inform decision-making in similar cases of missed posterior fracture-dislocation with an RHSL.

### Prognosis and risk of complications

Prognosis in posterior fracture-dislocations generally depends on time to diagnosis, RHSL size and morphology, and the presence of a PHF. Thus, missed or delayed diagnosis and larger defects are typically associated with poorer outcomes (1, 6). Even with joint-preserving ORIF, postoperative function may be difficult to predict because of complications; humeral head AVN is a key concern (5).

In this case, the patient initially underwent internal fixation; however, early postoperative imaging demonstrated persistent posterior dislocation. After revision surgery, the posterior dislocation was reduced, and fracture stability was restored with concomitant management of the RHSL. This case suggests that when the initial operation fails to reduce the dislocation and postoperative imaging shows persistent glenohumeral incongruity, early targeted revision may be warranted. Revision should address the bony defect and stabilizing structures in conjunction with reduction, which may reduce long-term uncertainty and preserve an opportunity for joint preservation and functional recovery in young patients.

Postoperative rehabilitation is equally important for functional recovery and complication prevention, as it requires a dynamic balance between early mobilization to prevent stiffness and avoiding excessive motion to prevent repair failure after shoulder dislocation surgery. This balance should be individualized according to injury type, surgical method, and patient characteristics. Previous studies have reported recurrence rates of shoulder dislocation of up to 70%. Strengthening exercises targeting internal rotation and adduction have been associated with a return to unrestricted activity in approximately 75% of patients, suggesting that rehabilitation should prioritize dynamic stability training to enhance neuromuscular control (17). However, when PHF is combined with dislocation and treated with plate fixation, rehabilitation must also balance bone healing with protection of the rotator cuff. A systematic review reported substantial variation in protocols, with sling immobilization ranging from 0 to 6 weeks, and patients treated with plate fixation often beginning rehabilitation earlier, initiating passive ROM at an average of approximately 2.2 weeks (18).

For RHSL associated with glenohumeral instability, a staged rehabilitation strategy may be warranted. From 0 to 6 weeks postoperatively, external rotation should be limited to within 30°, and combined movements of internal rotation, adduction, and extension that may provoke redislocation should be avoided. After 12 weeks, eccentric exercises and progressive strengthening can be introduced gradually, with attention to avoiding excessive deltoid loading to reduce the risk of acromial stress fracture. In addition, neuromuscular electrical stimulation may serve as an adjunct to improve external rotation function (19, 20). In this case, the arm was immobilized in 30° of abduction and 30° of external rotation for 3 weeks, followed by progressive passive ROM and advancement to active exercises; gradual weight-bearing began after 6 weeks. This protocol aligns with the rehabilitation rationale of restricting high-risk movements early while avoiding prolonged immobilization. At 5 years of follow-up, fracture union was achieved and ROM had recovered satisfactorily; the ASES score improved from 81 at 1 year to 90 at final follow-up, with no imaging evidence of AVN or post-traumatic arthritis. Nevertheless, current rehabilitation practice has two major limitations: first, most protocols are based on expert opinion rather than high-quality evidence; second, consensus is lacking on return to sport in young patients. Future randomized controlled trials are needed to determine optimal rehabilitation strategies, particularly regarding the safety of early mobilization and evaluation of home-based rehabilitation.

## Patient perspective

The patient reported that shoulder pain and functional limitation persisted after the initial operation and that he was concerned about the abnormal shoulder position. After revision surgery and rehabilitation, he noted progressive recovery of motion and strength and was satisfied with the long-term outcome, reporting full return to daily activities without pain at the final follow-up.

## Conclusions

PSD is highly prone to being missed at initial presentation. When PSD is combined with a PHF, severe pain often precludes acquisition of standard radiographic views, and accurate CT interpretation requires substantial subspecialty expertise. Clinicians should therefore remain vigilant; for patients with high-risk mechanisms (e.g., seizures, electrical injury, or high-energy trauma), a heightened level of suspicion and thorough imaging assessment are warranted to reduce missed diagnoses. In young patients with at least moderate RHSL, early joint-preserving management—focused on anatomic fracture reduction, defect reconstruction or buttress fixation, and repair of stabilizing structures—may achieve satisfactory long-term outcomes while avoiding arthroplasty.

## Data Availability

The original contributions presented in the study are included in the article/Supplementary Material, further inquiries can be directed to the corresponding authors.
